# A novel germline HAVCR2 (TIM-3) compound heterozygous mutation is related to hemophagocytic lymphohistiocytic syndrome in EBV-positive peripheral T-cell lymphoma (NOS) with down-regulated TIM-3 signaling

**DOI:** 10.3389/fonc.2022.870676

**Published:** 2022-09-23

**Authors:** Yang Zhang, Zhihua Wang, Guoyu Hu, Jieping Li, Yongheng Chen, Yi Jiang, Haiying Zhong, Xianling Liu, Chunhong Hu, Honglin Peng, Yunxiao Xu, Zhao Cheng, Guangsen Zhang

**Affiliations:** ^1^ Department of Oncology, The Second Xiangya Hospital, Central South University, Changsha, China; ^2^ Department of Hematology, The Second Xiangya Hospital, Central South University, Changsha, China; ^3^ Department of Hematology, The Affiliated Zhuzhou Hospital of Xiangya Medical College, Central South University, Zhuzhou, China; ^4^ Department of Hematology, The Central Hospital of Changsha City, Changsha, China; ^5^ Laboratory of Structural Biology, Key Laboratory of Cancer Proteomics of Chinese Ministry of Health, Xiangya Hospital, Central South University, Changsha, China; ^6^ Department of Pathology, The Second Xiangya Hospital, Central South University, Changsha, China

**Keywords:** HAVCR2 (TIM-3), mutation, hemophagocytic lymphohistiocytic syndrome, EBV-positive, peripheral T-cell lymphoma

## Abstract

Recently, it have been reported that Hepatitis A Virus-Cellular Receptor 2(HAVCR2,encoding T-cell immunoglobulin and Mucin-Containing Protein 3[TIM3]) mutations are associated with severe hemophagocytic syndrome(HLH) in subcutaneous panniculitis-like T-cell lymphoma(SPTCL),and there are also frequent mutations in sporadic SPTCL, suggesting the individuals harboring HAVCR2(TIM-3) germline mutations are highly susceptible to familial or sporadic SPTCL. Here, we identify a novel germline compound heterozygous mutation of TIM-3 gene,c.245A>G (p.*Tyr82Cys*) and c.265C>T(p.*Arg89Cys)* variations in a single familial case with EBV-positive peripheral T-cell lymphoma(NOS),accompanied HLH;we also detected *Tyr82Cys* germline mutation in TIM-3 gene in one sporadic patient with cutaneous T cell lymphoma. We screened the distributive frequencies for TIM-3 mutations in healthy controls(n=87), B-(n=79) or T-cell lymphoma(n=25) not SPTCL, and the results showed that the mutation was found in two out of 25 patients with T-cell lymphoma but was not detected in 79 patients with B-cell lymphoma nor in a group of 87 controls. The mRNA expression of TIM-3 on primary cells and transfected HEK293 cells reduced significantly, indicating *Tyr82Cys* and *Arg89Cys* mutations is a loss-of function mutations on TIM-3,resulting in a weakened TIM-3 signaling. Our results suggest *Tyr82Cys* TIM-3 germline mutations are not only limited in SPTCL, and also occurred in other types of T-cell lymphoma, especially complicated HLH. TIM-3 mutations may be an predisposing factor for T-cell lymphoma and molecular marker for auxiliary diagnosis in T cell lymphoma,especially complicated with HLH.

## Highlights

(1) A novel *HAVCR2* compound heterozygous mutations in germline is identified in EBV-positive peripheral T-cell lymphoma(NOS) with down-regulated expression on TIM-3 signaling.

(2) *HAVCR2* mutations are not only limited in SPTCL, but also could been found in other types of T-cell lymphoma, suggesting HAVCR2 mutation is a genetic predisposing factor for T-cell lymphoma.

## Introduction

Subcutaneous panniculitis T-cell lymphoma(SPTCL) is an uncommon subtype of T cell non-Hodgkin lymphoma mainly affecting younger individuals and with a female bias (1–3). The SPTCL patients typically manifest multiple subcutaneous nodules, systemic B-cell symptoms, and ~20% of cases complicated with hemophagocytic lymphohistiocytosis (HLH),which often yields a poor prognosis (1, 4). Several recent studies have identified germline homozygous or compound heterozygous mutations on Hepatitis A Virus-Cellular Receptor 2(HAVCR2) gene, encoding T-cell immunoglobulin and mucin domain-containing protein 3(TIM-3) in 59% of familial SPTCL (5),85% of sporadic Asian patients (6),and 25% of sporadic French cases (6),suggesting HAVCR2(TIM-3) germline mutations were susceptible to familial or sporadic SPTCL, and were associated with HLH/SPTCL. However, because there are larger differences between different race on the frequency of distribution of germline mutation of HAVCR2,and the specificity of HAVCR2 mutations for SPTCL molecular marker is still poorly understood. In the present study, we report a novel compound heterozygous mutation(*Tyr82Cys* and *Arg89Cys*) in TIM-3 gene in a familial Chinese patient with EBV-positive peripheral T-cell lymphoma(NOS) accompanied HLH, and the molecular structural characteristics of the mutation were analyzed. To elucidate if HAVCR2 mutations are only limited in SPTCL patients or if there is heterogenicity in various lymphoma types, we explored the change and significance of HAVCR2 mutations in T-cell or B-cell lymphoma.

## Methods

### Patients and samples

In this study, total of 104 cases (male:59;female:45; median age:51.25 years) with T-cell or B-cell lymphoma(including one patient with EBV-positive peripheral T-cell lymphoma(NOS) complicated with HLH, one patient with cutaneous T cell lymphoma(CTCL) were enrolled in the study. The 87 of healthy individuals were set as normal controls (male:27;female:60;median age:28.72 years). All samples were collected with informed consent and the study was approved by the Ethical Committee at the Second Xiangya Hospital, Central South University, Hunan, Changsha, China.

### Case reports

#### Case 1 and her family members

**Figure 1:**
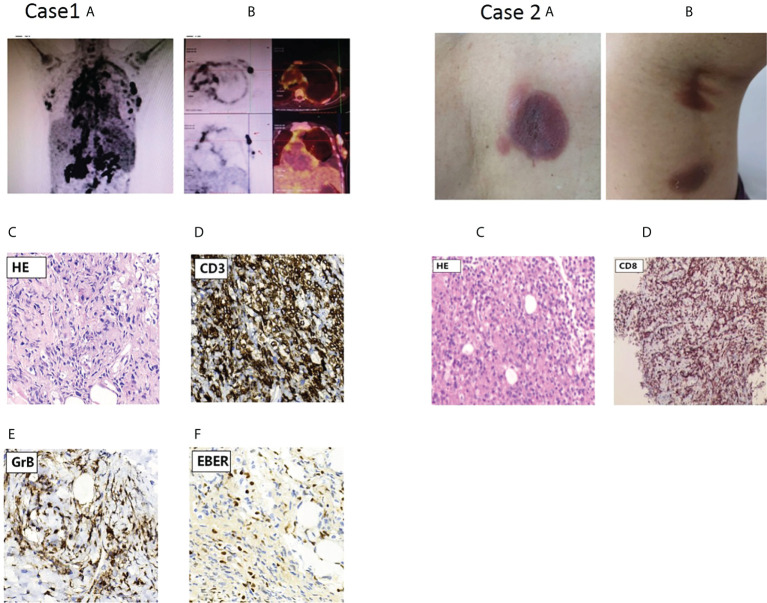
PET scan results of the proband in case 1 and skin lesions of case 2 with cutaneous T cell lymphoma(CTCL).Case1 **(A)** PET scan showing systemic Lymph node enlargement in upper and lower diaphragm and bilateral breast with Lymph node involvement; **(B)** PET scan pictures of different section indicated involved left breast lymph node with increased FDG metabolism, and arrow points to Hyper-FDG foci and the site of puncture on lymph node. **(C-F)** Representative pathological and immunohistochemical staining images of EBV-positive peripheral T-cell lymphoma(NOS);c.On H&E(hematoxylin & eosin staining);d,e,f, indicated positive immunohistochemical staining for CD3,GrB and EBER,respectively. Case2: characteristic skin lesions of the patient with CTCL harboring Y82C heterozygous mutation in TIM-3 gene; **(A)** localized erythema with ulcerated hollow in the left back; **(B)** two erythema with subcutaneous nodules in left thigh posterior. **(C)** Primary cutaneous CD8+ T-cell lymphoma: histopathology of section at H&E staining; **(D)** the tumor cells were positive for CD8.

**Figure 2:**
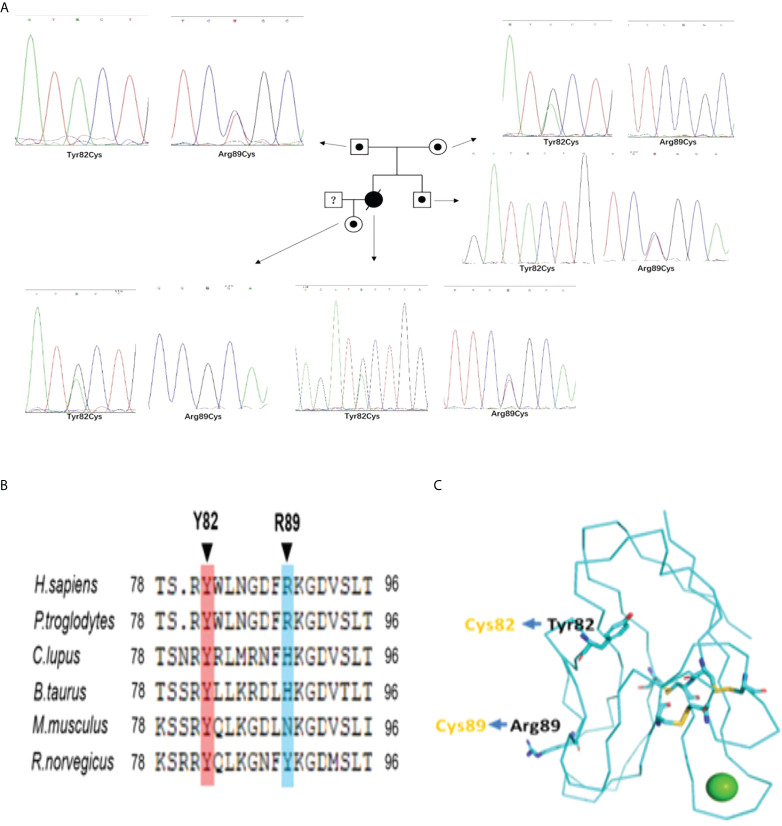
**(A)** The Pedigree diagram of the proband and corresponding sequencing results: Circles indicates female family members, squares indicates male family members, black small circle indicates singlely heterozygous mutation in TIM-3 gene; an black oblique line denote died of proband; Sanger sequencing pictures corresponding to the proband (Y82C and R89C compound heterozygous mutations) and her familial members(with singlely heterozygous mutation) in TIM-3 gene;1. **(B)** Sequence alignments in different species indicating homology of the mutated residues in TIM-3 protein;1. **(C)** Structure of the human TIM-3 IgV domain indicates the sites of the misfolding substitutions at Tyr82 and Arg89,and lead to TIM-3 protein misfolding aggregation(Protein Data Bank ID: 6DHB).

A 37-year-old woman was referred to our department for irregular fever and left chest and back pain in April,28 of 2020. She complained of an irregular fever (temperature:38^0^C-39.5^0^C) with chilly and fatigue. Gradually, her fever became continuous with left chest and back pain. Her white blood cells(WBC) was 2.64×10^9^/L. Prior to this evaluation, a visit to another hospital in Changsha city showed that chest and abdomen CT indicated bilateral pleural effusion with a small amount of pericardial effusion, hepatosplenomegaly with infarction of spleen,multiple lymphadenopathy in hilar and retroperitoneal area. Pathogen macrogenome detection showed EB virus infection. The bone marrow aspirate indicated a hypercellular marrow with significantly vacuoles in granulocytes; few phagocytic reticular cells were observed. The histopathologic diagnosis of right cervical lymph node biopsy histopathologic diagnosis was EBV positive lymphoproliferative disorders, immunohistochemical staining showed CD3+,CD30+,CD15-,CD21+,CD68+,EBER+;Monoclonal rearrangement of TCR-β was positive;NK cells activity was 14.32%(normal range:≥15.11%);sCD25 was 34,133pg/ml(normal range:<6400pg/mL). The whole exome second generation sequencing for genetic diseases (20,000 genes) results identified a compound heterozygous mutation of HAVCR2 gene(c.245A〉G,p.(*Tyr82Cys*) and c.265C〉T, p.(*Arg89Cys)*,and the mutations were confirmed by Sanger sequencing (**Figure 2A**). The patient’s past medical history was unremarkable and there was not any signs and symptoms involving in multiple subcutaneous nodules with multiple erythematous ulcerated plaques, and autoimmune disorders. At presentation, her physical examination showed mild anemia, superficial lymph nodes were not touched, hepatomegaly(below the inferocostal 5cm,splenomegaly (below the inferocostal 8 CM). Laboratory data showed hemoglobin of 108 g/L, WBC count of 4.28×10^9^/L and platelet count of 91×10^9^/L;Liver function showed an increased alanine aminotransferase(ALT:90.3u/L) and decreased albumin(32.8g/L);blood LDH 961.9u/L; triglyceride was 4.32mmol/l(Ref:<1.71mmol);Blood coagulation parameters showed decreased fibrinogen concentration(0.60g/L) and prolonged prothrombin time(PT 17.00sec);significantly increased ferritin (13223.81ng/ml); Lupus parameters showed anti-SSA+, anticardiolipin antibody IgG/IgM+;cytokines assay indicated increased IL-6(48.34pg/mL),IL-10(448.97pg/mL, and IFN-γ(22.26pg/ml) levels; EBV-DNA increased to 5.11×10^4^copies/ml. Positron emission tomography/computed tomography(PET-CT) scans results showed the nodules with abnormal increase of glucose metabolism in bilateral breast(**Figures 1 Case1A, B**) and multiple lymphadenopathy with Increased glucose metabolism up and down the diaphragm, suggesting lymphoma. According to PET-CT image, the patient accepted needle biopsy at right breast mass (**Figure 1B**), and histopathologic/immunohistochemical staining results(CD3^+^,CD4^+^,CD7^+^,CD8^+^,CD5^+^,GrB^+^,EBER^+^,LCA+Ki-67(70%),CD56^-^), indicated T-cell lymphoma (**Figures 1C–F**). The patient was diagnosed as EBV-positive peripheral T-cell lymphoma(NOS) complicated by hemophagocytic lymphohistiocytic syndrome(HLH),and given the therapies against HLH including dexamethasone, etoposide, ruxolitinib, and supporting/symptomatic treatment. Her symptoms once ameliorated. However, three week of after admission, the patient’s condition became ingravescence with constant high fever, chest tightness, tachyarrhythmia and cytokinemia. Although multiple treatment measures were employed, she died of severe acid-base imbalance and respiratory and circulatory failure on May 21, 2020.

#### Case 2

A 73-year-old woman was referred to our outpatient department of Hematology on June 29,.2020 for multiple subcutaneous nodules with russetishly erythematous plaques on the skull, trunk and extremities. Her whole course was half year and lacked systemic B symptoms. Physical examination revealed that there were multiple erythematous-like skin lesions with nodules distributing on skull, left back (**Figure 1 Case 2A**) and left thigh posterior (**Figure 1 Case 2B**) and enlarged lymph node(0.5cm×3.0cm) on left groin. Her liver and spleen not enlarged. The dermatopathology examination showed that the dermis contained abundant of lymphocytes and infiltrate with disseminated or nodular manner, suggesting lymphoma. The Pathological biopsy of subcutaneous nodular on left posterior thigh showed fibroplastic proliferation with more lymphocyte invasion; lacked tumor cells rim fat spaces (**Figure 1 Case 2C**),suggesting cutaneous T cell lymphoma(CTCL);immunohistochemical staining indicated CD3^+^, CD4^-^,CD8^+^,CD5^+^,CD7^+^,TIA1^+^,Ki-67(20%),CD56^-^,EBER^-^; tending to primary cutanenous CD8^+^ T-cell lymphoma (**Figure 1 Case 2D**). Laboratory data showed: routine blood test was normal; liver and kidney functions were in normal range; mildly increased LDH(286.5u/L); lupus parameters were negative; immunoglobulin G,A,M was normal. Analysis of T- cell subsets showed that CD4+ cells percentage(21%) was lower than normal limit (31%-60%); CD4+/CD8+ ratio was 0.59(normal range:0.80-2.75);TCR gene rearrangement indicated TCR-γ rearrangement. TIM-3 cDNA sequencing (peripheral blood and germline) indicated a heterozygous mutation (*c.245A〉G,p.(Tyr82Cys))*. her familial members refused the examination for *Tyr82Cys* mutation. Because pathological lesion only limited to skin and there was not systemic B symptoms,the patient refused further treatment, and followed-up at regular intervals.

### Methods

#### Extraction of DNA and whole exon sequencing

Genomic DNA was extracted from freshly peripheral blood mononuclear cells (PBMNCs) in the patients with T-cell or B-cell lymphoma (n=104) and oral mucosa cells(case 1 and her familial members and case2).For WES, only case 1 was carried out.Whole-exome capture was accomplished based on liquid phase hybridization of sonicated genomic DNA to the bait cRNA library, which was synthesized on magnetic beads using the SureSelectHuman All Exon V5 kit (Agilent Technology), according to the manufacturer’s protocol. Captured targets were subjected to massively parallel sequencing using Illumina high-throughput sequencing (HiSeq2000) with the paired-end 100 bp read option, according to the manufacturer’s instructions. Sequencing coverage was (20×) 99.59% and average sequencing depth was 129.07×.The sequencing procedure and result analysis were performed by KINGMED CENTER FOR CLINICAL LABORATORY, Changsha, Hunan, China).

#### Sanger sequencing

Primers flanking exon 2 of the TIM-3 gene were synthetized and used to validate the mutations identified by WES on genomic DNA and screen the mutations from all the patients with T-cell or B-cell lymphoma. The purified PCR products were bidirectional sequenced on an ABI3730 XL DNA sequenator. Primers sequence and PCR conditions are available upon request.

#### Plasmid construction and HEK293 cells transfection

HAVCR2-mutant ((NM_032782(Tyr82Cys&Arg89Cys), 957bp) and wild-type HAVCR2(NM_032782) (957 bp) were amplified by RT-PCR from entry clone. Amplification products were purified by the Wizard SV Gel and PCR Clean-Up System (Promega) and verified by Sanger sequencing. Purified PCR products were cloned into the GV658 vector (KpnI/PacI). The plasmid construction were performed by Ji-Kai Genechem Co., Shanghai, China. These plasmid DNAs were used to transfect HEK293 cells. Lipofectamine 2000 Reagent (Invitrogen) were used for transfection. Final DNA (500ng) and Final Lipofectamine 2000 (1ul) were used. Transfected HEK293 cells were grown in 24-well plates in DMEM (Gibco-BRL, USA) supplemented with 10% FBS. When cells were 80% confluent, further experiments were carried out.

#### Real-time quantitative PCR assay for TIM-3 mRNA expressions

Total RNA were extracted from peripheral blood mononuclear cells (PBMNCs) including the proband and her family members, three sporadic T-cell lymphoma and three healthy controls, or transfected HEK293 cells using Trizol reagent (Invitrogen,Carlsbad, CA, USA). Then 2μg RNA was reverse-transcribed into cDNA using the Revert Aid First Strand cDNA synthesis kit (Fermentas, Vilnius, Lithuania).Transcribed cDNA was amplified using a SYBR Green IPCR reagent (Takara, Shiga, Japan). Primers for TIM-3 gene were synthesized by the Bo-Shang Biotechnology Company, Shang Hai, China. The primers sequence are TIM3 Forward Sequence 5’- GACTCTAGCAGACAGTGGGATC-3’ Reverse Sequence 5’- GGTGGTAAGCATCCTTGGAAAGG-3’; β-actin Forward primer 5’-TTCCAGCCTTCCTTCCTGGG-3’ Reverse primer 5’- TTGCGCTCAGGAGGAGCAAT-3’.Relative expression quantification were applied to determine the levels of the target genes TIM3, which was normalized to that of β-actin(housekeeping gene) using 2 (-Δ Δ C(T)) method.

#### Bioinformatics assay and molecular model of the *Tyr82Cys* and *Arg89Cys* mutant

TIM-3 mutations were assessed by bioinformatics analysis using SIFT (Sorting Intolerant from Tolerant) and PROVEAN (Protein Variation Effect Analyzer) identified 2 nsSNPs to be putatively damaging/deleterious in at least one of the two tools used (7–9). Molecular modeling of *Tyr82Cys* and *Arg89Cys* mutants was carried out using SWISS-MODEL (10). Structure of the human TIM-3 IgV domain (Protein Data Bank ID: 6DHB) was used as a homology model (11).

### Statistical analyses

Quantitative data are presented as the mean ± standard error of mean (SEM). Comparisons between groups were made with one-way ANOVA or Student-T test. Statistical differences were considered significant at *P*< 0.05.

## Results

### Clinical features, TIM-3 variant pattern and outcome of case 1 and 2

In case 1 and her familial members, we identified compound heterozygous mutations in TIM-3 gene, which encodes T cell immunoglobulin mucin 3(TIM3),with *c.245A〉G,p*.*(Tyr82Cys)* and *c.265C〉T, p.(Arg89Cys)*.The compound heterozygous variants are different from those of reported at the second mutant point (5, 6, 12). For the evaluation of the mutations in the proband’s familial members, we confirmed that the proband’s mother and father harbored heterozygous *Tyr82Cys* or *Arg89Cys* mutation, respectively; Furthermore, the proband’s older brother and daughter also existed in heterozygous *Arg89Cys* and *Tyr82Cys* variant separately (**Figure 2A**), indicating TIM-3 mutations and transmission accord with autosomal recessive inheritance pattern (**Figure 2A**). Meanwhile, we also observed that these mutations both involved in somatic cells(PBMNCs) and germlin cells(oral mucosal cells).In case 2,the patient presented with multiple subcutaneous purple red plaques, localized in the extremities and trunk, with central collapse, and lack of B-cell symptoms. Her histopathologic, immunohistochemical and T-cell receptor rearrangement(TCR-γ) supported the diagnosis of cutaneous T cell lymphoma.Sanger sequencing indicated the patient harboring a heterozygous *Tyr82Cys* mutation in TIM-3 gene both involved somatic and germline cells.

### Distribution and frequency of TIM-3 mutations in T-,or B-cell lymphoma

In order to determine whether TIM-3 mutations were specific to T-cell lymphoma, we randomly chosen 104 of patients(including case1 and case2) with lymphoma (B-cell lymphoma: 79,T-cell lymphoma: 25 where 4 cases complicated with hemophagocytic lymphohistiocytosis),and evaluated the incidence rate of *Tyr82Cys* and *Arg89Cys* mutations in somatic cells. The results showed that 2 cases (Case1 and 2) existed in the mutations in TIM-3 gene on 25 cases with T-cell lymphoma; the incidence of TIM-3 mutations was 8.0%(2/25).Compared with healthy individuals(n=87),*Y82C* heterozygote in T-cell lymphoma was significantly enrichment. No *Tyr82Cys* or *Arg89Cys* mutations were found in 79 cases with B-cell lymphoma. Consequently, total incidence rate for TIM-3 gene mutations was 1.92%(2/104) in B- and T-cell lymphoma. As the distributive frequency in the general population, The Exome Aggregation Consortium (ExAC) showed the mutation frequency of *Tyr82Cys* in TIM-3 gene is 0.0035,while *Arg89Cys* mutation frequency is only 0.00002471,suggesting *Arg89Cys* mutation is very rare. In our investigation, 87 of healthy individuals were detected for TIM-3 variants, the result indicated that the TIM-3 mutations were negative(0/87),suggesting a heterozygous *Tyr82Cys* or *Arg89Cys* mutation observed in our patients is not a common polymorphism, but a related mutation that could possibly underlie T cell lymphoma.

### Homology of mutated residues and protein-folding assays of *Tyr82Cys* and *Arg89Cys* mutant

In our cases, the proband harbored *c.245A〉G*(*p.Y82C*) and *c.265C〉T(p.R89C*) mutations where*Y82C* was a highly conserved residues among species (**Figure 2B**),suggesting this mutation was deleterious(Provean prediction score:-8.45),but the conservatism of *R89C* at different species was lower (Provean prediction score:-2.29) (**Figure 2B**).Although in silico analysis showed *p.Arg89Cys* variation was predicted to be neutral,protein-folding assay showed *R89C* variation also plays important role in TIM-3 protein misfolding. The structure of the human TIM-3 IgV domain is stabilized by three disulfide bonds between *Cys38* and *Cys110*, *Cys52* and *Cys63*, and *Cys58* and *Cys109*.The two mutations, *Tyr82Cys* and *Arg89Cys*, might form unnatural intramolecular and/or intermolecular disulfide bonds, leading to protein misfolding or protein aggregation(**Figure 2C**). The misfolding of TIM-3 may eliminate the expression of TIM-3 on plasma membranes, and lead to continued immune activation, as well as increase the release of inflammatory cytokines, including TNF-α,IL-1β (5, 6).

### TIM-3 mutations weakens TIM-3 mRNA expression

**Figure 3:**
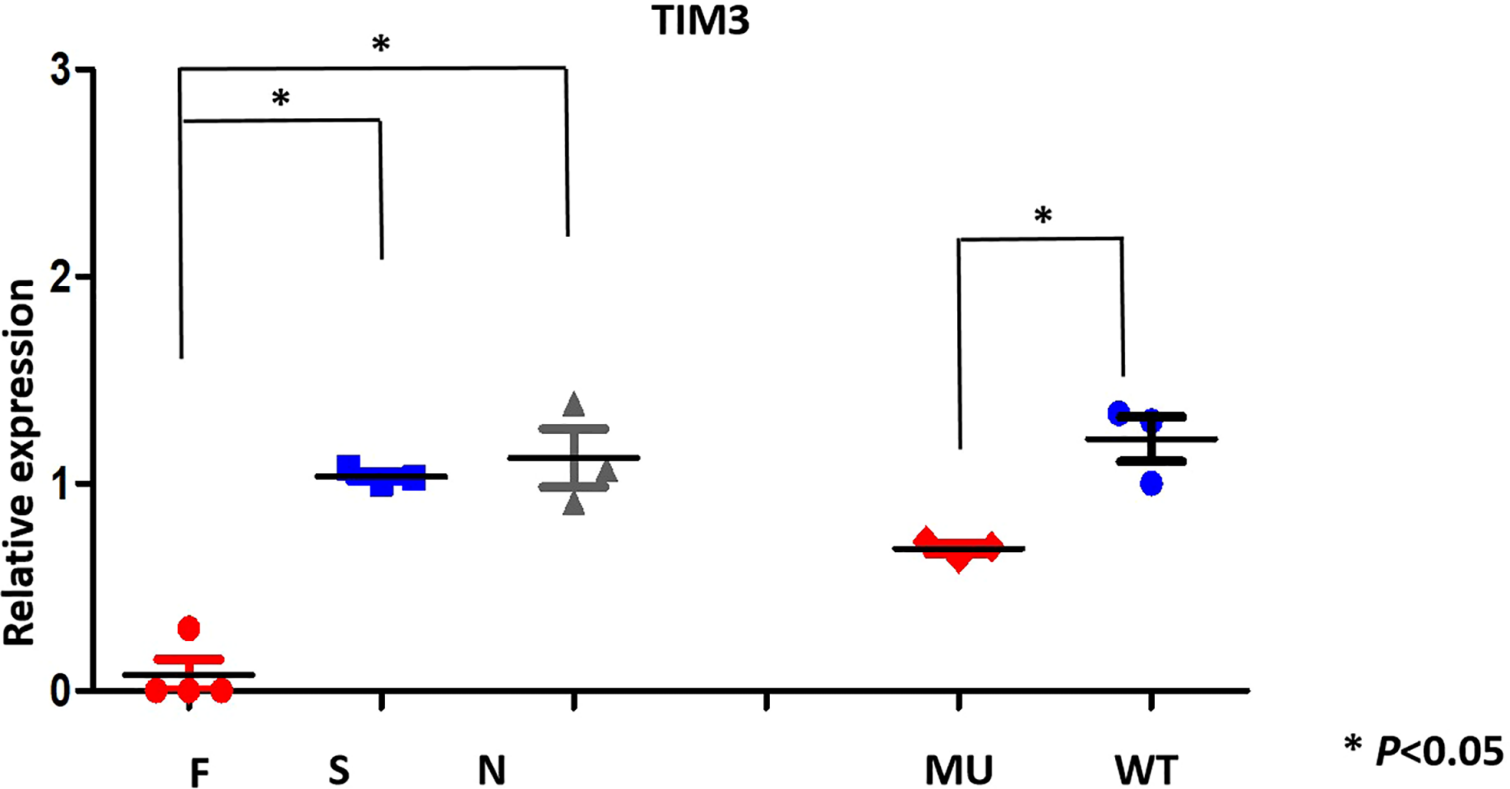
TIM-3 mRNA expression pattern (qRT-PCR) in family members(F) and proband; sporadic T -cell lymphoma(S), normal individuals(N) and transfected HEK293 cells.

To assess the effects of TIM-3 mutations on TIM-3 mRNA expression, we performed real-time quantitative PCR (RT-qPCR) on the primary cells from the patient and her familial members, as well as transfected HEK293 cells. RT-qPCR results showed that TIM-3 mRNA in four cases who harbored TIM-3 mutations, including familial 1’s proband and her familial members, was significantly lower than that of three sporadic T-cell lymphoma cases or three healthy controls (**Figure 3**); In the corresponding HEK293 cells loading TIM-3 mutant, the TIM-3 mRNA expression was also significantly downregulated, suggesting the *Tyr82Cys* and *Arg89Cys* mutation in TIM-3 gene is a loss-of -function mutation.

## Discussion

SPTCL is pathologically diagnosed based on CD8+ neoplastic infiltration and TCR α/β+ T cells infiltrating into subcutaneous tissue. Recently, a few of documents indicate that SPTCL is a genetically distinct disease with highly frequent HAVCR2(TIM-3) gene mutation (5, 6, 12).It have been shown that HAVCR2 mutations happened to 59% of familial SPTCL (5) and 85% of sporadic Asian patients (6). However, the occurrence of the HAVCR2 mutations and the specificity on lymphoma classification or clinical features have not yet been fully elucidated.

In the present studies, we for the first time identified that a compound heterozygous mutations([*Tyr82Cys*]+[*Arg89Cys*+] in TIM-3 gene existed in one patient with EBV-positive peripheral T-cell lymphoma(NOS),instead of SPTCL, as well as had family gathering tendency for TIM-3 mutation. The compound heterozygous mutations were not different from reported of that at the second mutant locus[*Arg89Cys*] (5, 6, 12),and may be responsible for the morbidity of T-cell lymphoma and attack of hemophagocytic lymphohistiocytic syndrome in the case. In case2,we also detected a single heterozygous mutation in TIM-3 gene(*Tyr82Cys*),this mutation points to germline origin. Curiously, the diagnosis of the patient was cutaneous T cell lymphoma not SPTCL, suggesting TIM-3 gene mutation is not only limited to SPTCL.As for the underlying genetic cause of SPTCL, remains unknown, but a familial predisposition has been reported (13). It is worth emphasizing that our case 1 did not manifest any evidences involved in subcutaneous nodules or plaques both clinical phenotypes and the skin images of PET-CT, indicating TIM-3 gene mutation also appeared in other types of T-cell lymphoma, including peripheral T cell lymphoma. In fact, the case 1 had diffuse lymph nodes and organ involvement, which is rare in SPTCL (4, 14),and her disease was rapid progression until death, reflecting a poor prognosis. It have been reported that if hemophagocytic syndrome(HLH) is present, the prognosis of SPTCL is very poor (15, 16),which is consistent with our case except for different subtypes of lymphoma. Recent literatures showed that HLH appears as a defining feature of SPTCL with TIM-3 mutation,and the incidence of TIM-3 mutations in HLH was 87.5% (5),17% (6) and 18.2% (12), respectively. The discrepancy in the frequencies of HLH in these groups may be associated with sample selection bias or ethnic background.

In case 1,the compound heterozygous mutations of TIM-3 come from her unaffected father and mother respectively, and her old brother and her daughter also existed in single heterozygous mutation on TIM-3 gene, but not involved in lymphoma onset (**Figure 2A**). As TIM-3 gene’s the second mutant locus(*Arg89Cys*),it is a novel variant and different from reported 3 cases with compound heterozygous mutations on TIM-3 gene in SPTCL(2 for *Ile97Met* (5, 12),; 1 for *T101I (* 6)).Although the homology of the mutant residues (*Arg89Cys)* was not highly conserved, the mutant locus is localized in the immunoglobulin-like domain (IgV-like domain) and the transmembrane domain for TIM-3 protein (5),suggesting *Arg89Cys* mutation plays important role in modulating immune functions and facilitating disease penetrance. Further, the structure modeling of the human TIM-3 IgV domain showed that the sites of the misfolding substitutions at *Tyr82(Y82*) and *Arg89(R89*), the extra a pair of disulfide bond(*82Cys* and *89Cys*), may form unnatural intramolecular and/or intermolecular disulfide bonds, leading to protein misfolding or protein aggregation.

A search for the mutation in 87 population-based healthy controls and 104 cases with B-,or T- cell lymphoma revealed that two cases had heterozygous mutations (1.92%) in the TIM-3 gene, furthermore, only limited in T-cell lymphoma; In healthy controls, the incidence of TIM-3 mutations was 0.00% (0/87),suggesting heterozygous mutations in TIM-3 gene are not a polymorphism,but a pathogenic mutation, as well as susceptible to T-cell lymphoma.

The TIM-3, as a negative checkpoint regulator, plays a critical role in innate immunity and inflammatory responses (17, 18).It have been shown that TIM-3 is able to inhibit effector T cell responses by decreasing interferon-γ(IFN-γ)-driven inflammation (17),and the defects of TIM-3 function may explain the HLH manifestations seen in TIM-3-mutant SPTCL (5). Accordingly, in our case1,we observed increased IL-6,IL-10,and IFN-γ levels, suggesting an Inflammatory activation, and constituting the feature of cytokine storm in HLH (19).

To reveal the mechanism of TIM-3 mutations for predisposing to T cell lymphoma, it is essential to evaluate the expression levels of TIM-3 mRNA and protein. We showed that TIM-3 mRNA expression in primary PBMNCs were significantly decreased in the proband and her family members than that of sporadic T cell lymphoma and healthy individuals with wild-type TIM-3, which is consistent with the mRNA expression of TIM-3 in the transgenic HEK293 cells, suggesting Tyr82Cys and Arg89Cys double mutation of TIM-3 is a loss-of -function mutation,and reduced TIM-3 expression may contribute to the pathogenesis of T cell lymphoma and immune imbalance of HLH.

In conclusion, we identify a familial T-cell lymphoma who is caused by compound heterozygous mutation in germline TIM-3,and shows an autosomal recessive-inherited pattern, as well as is associated with EBV-positive peripheral T-cell lymphoma(NOS),not SPTCL. A novel *p. Arg89Cys* and *p.Tyr82Cys* mutations in TIM-3 gene is clearly pathogenic mutation, and is responsible for the amplified Inflammatory signalings and HLH attack. The prevalence of TIM-3 mutations in our study is not only limited in SPTCL, it is also seen in other types of T-cell lymphoma. T-cell lymphoma complicated HLH should be screened routinely for TIM-3 mutations.

## Data availability statement

The raw data supporting the conclusions of this article will be made available by the authors, without undue reservation.

## Ethics statement

All samples were collected with informed consent, and the study was approved by the Ethical Committee at the Second Xiangya Hospital, Central South University, Hunan, Changsha, China. The patients/participants provided their written informed consent to participate in this study. Written informed consent was obtained from the individual(s) for the publication of any potentially identifiable images or data included in this article.

## Author contributions

GZ diagnosed and treated the patients, designed the study and composed the paper. YZ and ZC performed main experiments; ZW and HZ performed partly experiments; GH, JL, XL, CH, HP, and YX helped with clinic sample collection and case-management. YC performed the protein structure prediction. YJ performed the pathological experiments. All authors contributed to the article and approved the submitted version.

## Funding

This work was supported by the National Natural Science Foundation, P.R. China (No.81500171); Hunan Province Natural Science Foundation (No.2019JJ40449) and the National Natural Science Foundation, P.R. China (No.81470323).

## Acknowledgments

We thank the patients, healthy blood donors, and clinical teams who have been involved in the study.

## Conflict of interest

The authors declare that the research was conducted in the absence of any commercial or financial relationships that could be construed as a potential conflict of interest.

## Publisher’s note

All claims expressed in this article are solely those of the authors and do not necessarily represent those of their affiliated organizations, or those of the publisher, the editors and the reviewers. Any product that may be evaluated in this article, or claim that may be made by its manufacturer, is not guaranteed or endorsed by the publisher.
